# Influence of Processing Parameters on Flow Behaviour of Ultra-Large-Section Beam Blank Continuous Casting Mould

**DOI:** 10.3390/ma18020275

**Published:** 2025-01-09

**Authors:** Yulong Deng, Nanzhou Deng, Jintao Duan, Yibo Li, Qi Gao, Weihua Ni, Jingquan Peng

**Affiliations:** 1 Light Alloy Research Institute, Central South University, Changsha 410017, China; 2 China National Heavy Machinery Research Institute Co., Ltd., Xi’an 710054, China; 3 College of Mechanical and Electrical Engineering, Central South University, Changsha 410012, China

**Keywords:** ultra-large-section beam blank, continuous casting, flow behaviour, numerical simulation

## Abstract

The complex cross-sectional shape of oversized beam blanks and the size effect of ultra-large-section beam blanks create severe issues related to the surface and internal quality of the castings. To ensure quality and control in the production of ultra-large-section beam blanks, a numerical and physical model of molten steel flow in the three-port submerged entrance nozzle (SEN) mould, with section dimensions of 1300 × 510 × 140 mm, was established. This model was created using numerical simulations and NSGA-II genetic algorithm optimisation, and the impact of the casting speed and SEN immersion depth on the mould’s flow behaviour was investigated. The results showed that a deeper SEN immersion depth resulted in, a greater impact depth of the molten steel, and the surface flow velocity decreased. Both the impact depth and the surface flow velocity of the molten steel increased with increasing casting speed. The physical simulation and numerical simulation of the molten steel flow form and flow velocity distribution in the mould were in good agreement with each other, thus verifying the accuracy of the numerical simulation. The process parameters derived from this study were all within an appropriate range, which can help to improve the quality of continuously cast beam blanks. This also provides guidance for selecting optimal parameters for actual continuous casting production processes.

## 1. Introduction

Continuous casting is an ideal method for producing beam blanks and can increase production, save costs, and reduce energy consumption. Compared with conventional small-section beam blanks, ultra-large-section beam blanks have a larger flexural modulus and higher structural strength [1,2,3,4]. However, the intricate cross-sectional geometry of oversized beam blanks, along with the size-dependent effects associated with large beam blanks [5] create non-uniform steel flow and cooling rates at different positions in the transition zone of the web and flange during continuous casting [6,7]. This makes them prone to cracking, resulting in major surface and internal quality issues [8,9].

The elimination of inclusions, heat transfer at the steel–slag interface, flow, and solidification are all greatly impacted by the flow behaviour of molten steel in a mould [10]. If not adequately controlled, the improper flow behaviour of molten steel in the mould during continuous casting can lead to the formation of various defects in beam blanks [11]. Thus, the appropriate flow behaviour and temperature distribution of molten steel within the mould play a crucial role in ensuring the uniform formation of the solidified shell in beam blanks, which is essential for maintaining their quality [12,13]. However, the flow dynamics of the molten steel inside the mould are influenced by the design of the submerged entry nozzle (SEN) when the mould has a specific cross-sectional shape [14]. The structure of the SEN significantly impacts the molten steel flow, heat transfer, solidification process, and the surface quality of the beam blanks [15,16,17].

Research has found that the commonly used straight-through single-port SEN has a greater impact depth on molten steel, which makes it difficult to remove inclusions and melt the protective slag [10,18]. The use of two-port SEN continuous casting ensures the symmetry of molten steel flow, which improves solidification in the mould [4,10]. However, the above two types of SENs are mainly used to produce traditional castings such as slabs with relatively simple geometric shapes. It is difficult to determine a reasonable SEN structure to control the quality of beam blanks with complex cross-sectional shapes, or even ultra-large cross-sectional beam blanks. To tackle this issue, a computational fluid dynamics (CFD) model was developed to simulate the solidification behaviour of molten steel within an ultra-large beam blank mould with dimensions of 900 × 510 × 130 mm. Based on the simulation results of two-port and three-port SENs, the numerical simulation results of the three-port SEN were combined with the non-dominated sorting genetic algorithm II (NSGA-II). Subsequently, the optimal range of structural parameters for the three-port SEN, which demonstrated reduced boundary layer separation and enhanced fluid dynamic performance, was identified to effectively control the continuous casting quality of ultra-large-section beam blanks [5].

Research has shown that optimising the SEN is an economically effective method to control the quality of castings [19]. In addition to the SEN structure, process parameters such as the SEN immersion depth and casting speed also affect the flow field inside the mould [10]. Reasonable process parameters improve the quality of castings and increase the production efficiency of continuous casting [20]. In recent years, the large-scale development of infrastructure sectors, including construction engineering, transportation, and marine engineering, has led to increasingly stringent demands regarding the size and performance of beam blanks [21]. Therefore, under a specific SEN structure, it is necessary to conduct research to determine the appropriate SEN immersion depth, casting speed, and other process parameters. It is essential to elucidate the influence of these factors on the flow field in order to effectively control the molten steel flow behaviour within the mould and facilitate the uniform growth of the solidified shell in the beam blank. This approach optimises the production efficiency of continuous casting [22].

In response to the optimal range of the three-port SEN structure determined by the NSGA-II genetic algorithm, this study established a model for an ultra-large-section beam blank mould with dimensions of 1300 × 510 × 140 mm and examined the impact of process parameters on the flow behaviour of the three-port SEN continuous casting mould. First, the effect of SEN immersion depth on the flow behaviour within the mould was investigated through numerical simulations, leading to the determination of an optimal SEN immersion depth. Second, with the immersion depth fixed, the influence of different casting speeds on the flow field behaviour inside the mould was analysed, followed by the identification of the casting speed that maximised production efficiency. Finally, a physical simulation was conducted to examine the flow field characteristics and velocity distribution within the mould under the two determined parameters. Numerical simulations were used to compare and validate the results in order to determine the optimal process parameters for the SEN structure. These results provide a valuable reference for the subsequent use of SEN-structured moulds in the continuous casting production of ultra-large-section beam blanks.

## 2. Mathematical and Physical Models

### 2.1. Mathematical Models

**Figure 1:**
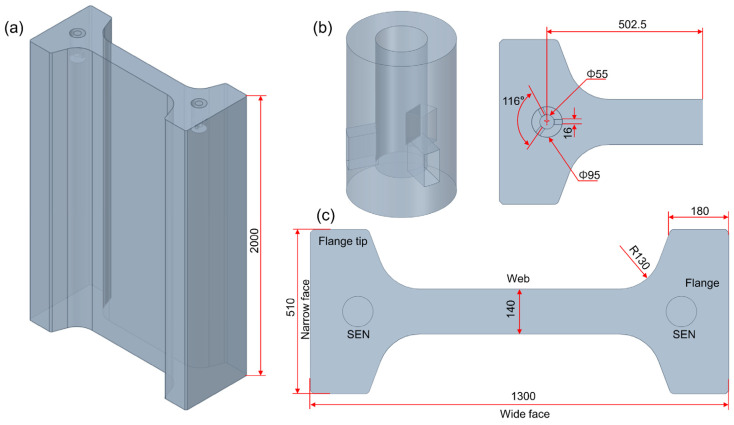
Schematic representation of the three-port SEN mould: (**a**) mould for the beam blank, (**b**) three-port SEN, and (**c**) cross-sectional view of the beam blank.

This study designed a three-port SEN mould for the shape characteristics of ultra-large-section beam blanks. The geometric structure, installation method, and beam blank cross-sectional shape of the mould are shown in Figure 1. The working length of the mould was 700 mm. To prevent potential backflow of fluid at the mould outlet during numerical simulations, the total length of the computational domain was extended to 2000 mm.

#### 2.1.1. Basic Assumptions

Due to the complex multi-physics-coupled metallurgical physical processes in the mould during actual continuous casting, the following assumptions were made in the formulation of the mathematical model:

(1) The molten steel is assumed to be an incompressible Newtonian fluid with constant viscosity in a steady-state condition [23].
(2) The influence of solidification shrinkage, protective slag, and mould vibrations on the flow and thermal behaviour of the molten steel is disregarded [24].
(3) The latent heat associated with solid-phase transformation is considered negligible in comparison to the latent heat of solidification.

#### 2.1.2. Governing Formulations and Physical Properties

A fluid flow model was developed to describe two primary flow types within the cast strand: interdendritic flow in the mushy zone and turbulent flow in the liquid core [23]. Based on the previously mentioned assumptions, a fundamental equation was derived to characterise the molten steel flow. The standard k−ε model was utilised to capture the turbulence effects within the cast strand [24]. The governing equations for the molten steel flow include the continuity equation, the Navier–Stokes equation, and the k−ε turbulence equations.

Continuity equation:

(1) $$\frac{\partial \rho }{\partial t}+\nabla \cdot \left(\rho \vec{u}\right)=0$$

where ρ denotes the density of the molten steel,$\;kg/m^{3}$; and u represents the fluid velocity, m/s.

Navier–Stokes equation:

(2) $$\frac{\partial \left(\rho u_{i}\right)}{\partial t}+\rho \frac{\partial \left(u_{i}u_{j}\right)}{\partial x_{j}}=-\frac{\partial P}{\partial x_{i}}+\frac{\partial }{\partial x_{j}}\left[\mu_{eff}\left(\frac{\partial u_{i}}{\partial x_{j}}+\frac{\partial u_{j}}{\partial x_{i}}\right)\right]+\rho g$$

(3) $$\mu_{eff}=\mu_{l}+\mu_{t}$$

(4) $$\mu_{t}+\rho c_{\mu }\frac{\varepsilon^{2}}{k}$$

where $u_{j}$ is the velocity of the fluid in the *j* direction, m/s; P is pressure, Pa; $x_{j}$ is the coordinate component in the j direction; $\mu_{eff}$ is the effective viscosity coefficient; $u_{l}$ is the laminar viscosity, kg/(m·s); $u_{t}$ is the turbulent viscosity, kg/(m·s); $c_{\mu }$ is an empirical constant, taken as 0.09; k is turbulent kinetic energy, $m^{2}/s^{2}$; ε is turbulent energy dissipation rate, $m^{2}/s^{3}$; g is gravitational acceleration, taken as 9.81 $m/s^{2}$.

k−ε turbulence equations:

(5) $$\frac{\partial \left(ku_{i}\right)}{\partial x_{i}}=\frac{\partial }{\partial x_{i}}\left(\frac{\mu_{eff}}{\sigma_{k}}\frac{\partial k}{\partial x_{i}}\right)+G_{k}-\rho \varepsilon$$

(6) $$\rho \frac{\partial (\varepsilon u_{i})}{\partial x_{i}}=\frac{\partial }{\partial x_{i}}(\frac{\mu_{eff}}{\sigma_{\varepsilon }}\frac{\partial \varepsilon }{\partial x_{i}})+C_{1\varepsilon }\frac{\varepsilon }{k}G_{k}-C_{2\varepsilon }\rho \frac{\varepsilon^{2}}{k}$$

(7) $$G_{k}=\mu_{t}\frac{\partial u_{j}}{\partial x_{i}}(\frac{\partial u_{i}}{\partial u_{j}}+\frac{\partial u_{j}}{\partial u_{i}})$$

where $G_{k}$ is the turbulent kinetic energy generation term; $C_{1\varepsilon }$, $C_{2\varepsilon }$, $\sigma_{k}$, and $\sigma_{\varepsilon }$ are empirical constants, using the recommended values of Launder and Spalding, set as $C_{1\varepsilon }$ = 1.44, $C_{2\varepsilon }$ = 1.92, $\sigma_{k}$ = 1.0, and $\sigma_{\varepsilon }$ = 1.3 [4].

The enthalpy–porosity method (EPT) was used to simulate the mushy zone (partially solidified region) as a porous medium. The percentage of the liquid phase in each computational cell was used to calculate its porosity. The porosity was set to zero in areas of complete solidification, which caused the velocity to stop there [11,25]. To compensate for the decreased porosity in the mushy zone, additional source terms $S_{p}$, $S_{k}$, and $S_{\varepsilon }$, were added to the momentum, turbulent kinetic energy (k), and turbulent dissipation rate (ε) equations, respectively.

(8) $$S_{p}=\frac{(1-f_{l}{)}^{2}}{f_{l}^{3}+\xi }\cdot A\cdot \left(\vec{u}-{\vec{u}}_{c}\right)$$

(9) $$S_{k}=\frac{(1-f_{l}{)}^{2}}{f_{l}^{3}+\xi }\cdot A\cdot k$$

(10) $$S_{\varepsilon }=\frac{(1-f_{l}{)}^{2}}{f_{l}^{3}+\xi }\cdot A\cdot \varepsilon$$

where $f_{l}$ refers to the liquid fraction; ξ is a small constant typically set to 0.0001 to avoid division by zero in floating-point calculations; A represents the mushy zone constant, valued at $1\times 10^{8}\;$ [25]; and *u_c_* indicates the casting speed, m/s.

A heat transfer and solidification model was developed, and the enthalpy–porosity technique (EPT) was employed to simulate the heat transfer and solidification processes of molten steel [26].

Energy conservation equation:

(11) $$\frac{\partial }{\partial t}\left(\rho H\right)+\nabla \left(\rho \vec{u}H\right)=\nabla \cdot \left(\lambda_{eff}\nabla T\right)+S_{h}$$

where $\lambda_{eff}$ is the effective thermal conductivity, calculated by adding the thermal conductivity λ and the turbulent thermal conductivity $\lambda_{t}$, with units of $W\cdot m^{-1}\cdot K^{-1}$, and T represents the temperature in Kelvin, K.

The total enthalpy is described as follows:

(12) $$H=h_{ref}+\int_{T_{ref}}^{T}C_{p}dT+f_{l}L$$

where $h_{ref}$ is the reference enthalpy, $J\cdot {kg}^{-1}\cdot {mol}^{-1}$; $T_{ref}$ is the reference temperature, K; $C_{p}$ denotes the specific heat, $J\cdot {kg}^{-1}\cdot K^{-1}$; and *L* represents the latent heat, $J\cdot {kg}^{-1}$.

A source term $S_{h}$ to describe the liquid–solid transition and the formation of the mushy zone is included in the energy conservation equation.

(13) $$S_{h}=\rho L(\frac{\partial (1-f_{l})}{\partial t}+u_{c}\nabla (1-f_{l}))$$

Equation (14) was employed to determine the liquid fraction:

(14) $$f_{l}=\frac{T-T_{s}}{T_{l}-T_{s}}$$

where $T_{l}$ and $T_{s}$ are the liquidus and solidus temperatures of the molten steel, K.

A geometric model was developed based on the cross-section of the mould and the structure of the SEN. The hexahedral mesh was generated using Ansys Meshing, and iterative calculations were carried out with Ansys Fluent v2024R1. The convergence criteria were set with an energy residual less than 10⁻⁶ and other residuals less than 10⁻⁴. Q235B carbon structural steel’s physical properties are essential for heat transfer, fluid flow, and solidification in the mushy zone. The material parameters relevant to the numerical simulation are presented in Table 1.

#### 2.1.3. Boundary Conditions

The mould inlet used the velocity inlet boundary conditions. The inlet velocity was obtained by converting the casting speed through the continuity equation based on the conservation of mass during continuous casting.

(15) $$v_{0}=\frac{v_{e}\times A_{out}}{A_{in}}$$

Equations (16) and (17) were used to determine the turbulent kinetic energy and dissipation rate.

(16) $$k=0.01v_{0}^{2}$$

(17) $$\varepsilon =\frac{2k^{1.5}}{d_{H}}$$

where $v_{0}$ is the inlet velocity, m/s; $v_{e}$ is the casting speed, m/s; $A_{out}$ is the cross-sectional area of the beam blank, $m^{2}$; $A_{in}$ is the cross-sectional area of the mould’s SEN, $d_{H}$ is the hydraulic diameter of the SEN, m.

To preserve mass conservation, the outlet was designated as a pressure outlet and the inlet temperature as the casting temperature. All other walls were modelled as zero-slip, with a velocity equal to the casting speed, while the meniscus was considered as an adiabatic surface with no shear force. The heat flux was calculated using the temperature differential between the cooling water’s entrance and output, assuming no energy loss occurred during the numerical simulations.

(18) $$\bar{q}=\frac{\rho_{w}C_{p}Q_{w}\Delta T}{S_{eff}}$$

where $\rho_{w}$ is the density of cooling water, $kg/m^{3}$; $C_{p}$ is the specific volume of cooling water, $J\cdot {kg}^{-1}K^{-1}$; $Q_{w}$ is the flow rate of cooling water, $m^{3}/s$; ΔT is the temperature difference between the cooling water at the inlet and the outlet of the cooling water, K; $S_{eff}$ is the effective contact area between the molten steel and mould wall, $m^{2}$.

### 2.2. Physical Models

#### 2.2.1. Water Model

A water model was established to simulate the flow behaviour inside a three-port ultra-large-section beam blank mould, with a geometric ratio of 1:1 between the model and prototype. According to similarity theory, to ensure a similar fluid flow inside the water model and prototype mould, it was necessary to make the dynamics of the water model and the prototype similar. Because the flow of molten steel is mainly dominated by gravity and inertial forces, as long as the Froude numbers of the water model and the prototype are equal, the dynamics should be similar, i.e., $Fr_{p}=Fr_{m}$. From this, Equation (19) can be derived [27]:

(19) $$\frac{v_{m}^{2}}{g_{m}L_{m}}=\frac{v_{p}^{2}}{g_{p}L_{p}}$$

where m is the index of the water model; p is the actual mould index; v is the flow velocity in the flow field, m/s; g is the gravitational acceleration, $m/s^{2}$; L is the characteristic length, m.

Similarity factor:

(20) $$\lambda =\frac{L_{m}}{L_{p}}$$

According to Equations (19) and (20), the conversion relationship between the water simulation test and prototype mould flow rate and volumetric flow rate can be calculated.

(21) $$v_{m}=\sqrt{\lambda }v_{p}$$

(22) $$Q_{m}={\lambda^{\frac{5}{2}}Q}_{p}$$

The dimensions of the cross-section of the beam blank were 1300 × 510 × 140 mm. According to the above formula, the water flow rate corresponding to different casting speeds in the mould can be calculated. Due to the continuous casting machine used by a certain steel plant to produce beam blanks with a cross-section of 1300 × 510 × 140 mm, the maximum casting speed is 0.60 m/min, the designed casting speed is 0.57 m/min, and the minimum casting speed is 0.47 m/min. Therefore, two corresponding casting speeds of 0.47 m/min and 0.57 m/min were set for the research. In order to maintain an equal difference between the two previous casting speeds, a research casting speed of 0.67 m/min was set. The calculated water model experimental fluxes for different casting speeds are shown in Table 2.

#### 2.2.2. Experimental Apparatus and Method

**Figure 2:**
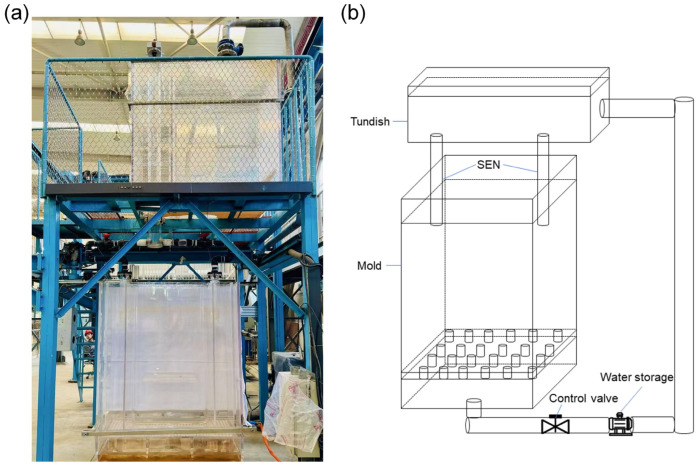
Experimental apparatus for physical simulation. (**a**) Photograph; (**b**) Schematic diagram.

A water model was established for the tundish and beam blank mould using transparent acrylic sheets in a 1:1 ratio. To eliminate the impact of reversed flow on the flow field within the mould, the model was extended to a length of 2000 mm, and the experimental apparatus is shown in Figure 2.

**Figure 3:**
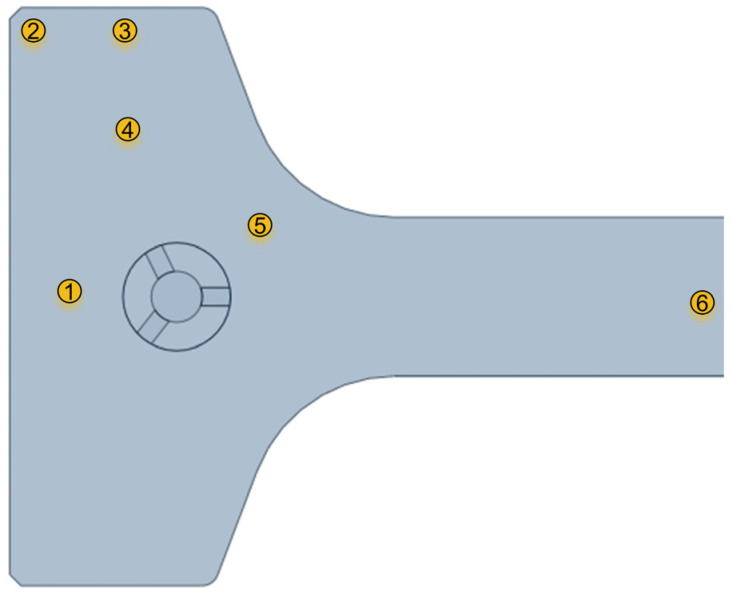
Selected characteristic points: the centre of the narrow face (1#), the outer corner of the flange (2#), the centre of the flange tip (3#), the centre of the flange (4#), the R corner (5#), and the centre of the web (6#).

To facilitate studying the flow of molten steel in the ultra-large-section beam blank mould, an LS300-A portable flow velocity metre was used to measure the horizontal surface flow velocity at six different characteristic points 20 mm from the free surface of the mould. The key characteristic points were located at the centre of the narrow face (1#), the outer corner of the flange (2#), the centre of the flange tip (3#), the centre of the flange (4#), the R corner (5#), and the centre of the web (6#). The selected cross-sectional characteristic points are shown in Figure 3. The same characteristic points were selected in the analysis of numerical simulation results.

## 3. Results and Discussion

### 3.1. Influence of the SEN Immersion Depth on Flow Behaviour

**Figure 4:**
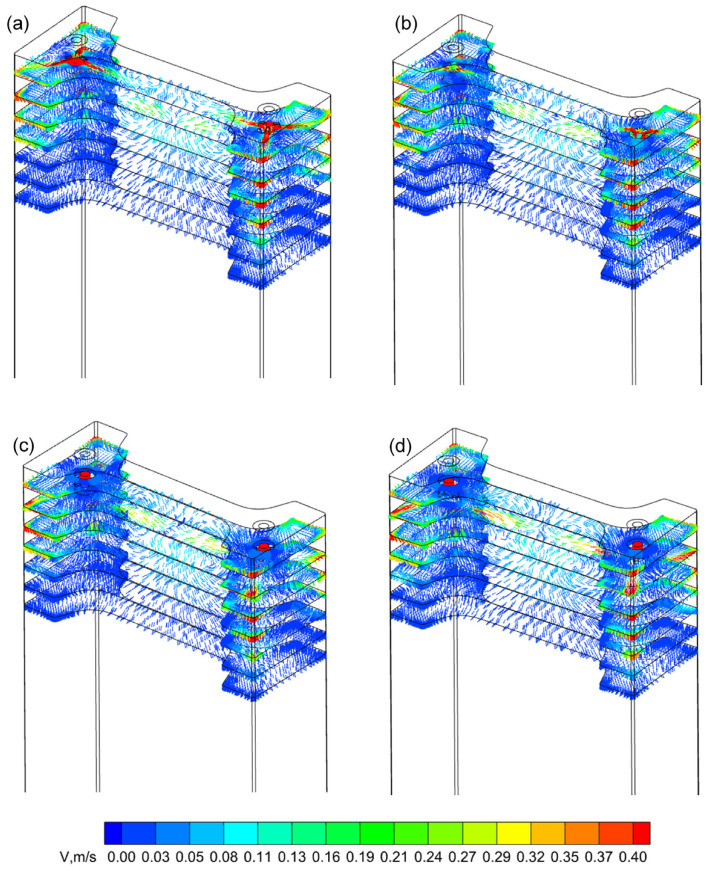
Velocity vector maps at a casting speed of 0.57 m/min and different immersion depths of the SEN: (**a**) 80 mm; (**b**) 100 mm; (**c**) 120 mm; (**d**) 140 mm.

The immersion depth of the SEN affects the impact depth and flow state of the molten steel in the mould, which in turn has a significant effect on the formation of the solidified shell of the beam blank and the removal of inclusions. Figure 4 shows, at a casting speed of 0.57 m/min, the velocity vector diagrams at depths of 100, 200, 300, 400, 500, 600, and 700 mm below the meniscus. In the three-port SEN mould, the molten steel flowed out laterally from the SEN and rushed towards the flange tip where it generated reflux that resulted in a higher flow velocity at the shallow flange tip of the mould. Due to the mutual impact of the two radial SENs flowing towards the centre of the web, a “self-braking” phenomenon occurred [28], which enhanced the stability of the steel flow in the web area. As the immersion depth increased, the impact depth of the molten steel in the three-port mould gradually deepened, but the impact depth of the three-port SEN-structured mould was relatively small overall. Figure 4 shows that the impact of the molten steel is more significant at 200 mm below the meniscus at different immersion depths of the SEN. At 300 mm below the meniscus, the impact is significantly reduced at immersion depths of 80 mm and 100 mm, while the impact is still significant at immersion depths of 120 mm and 140 mm. Compared to the impact depth of the single-port SEN studied in the literature [1] at an immersion depth of 70 mm and a casting speed of 0.57 m/min, the impact depth of the three-port SEN molten steel at an immersion depth of 140 mm is still relatively small.

**Figure 5:**
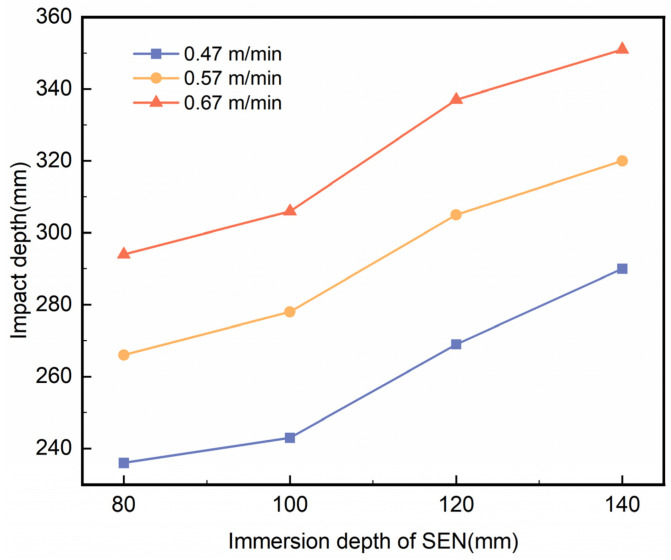
The relationship between the variation in molten steel impact depth and the immersion depth of the SEN.

Figure 5 shows the relationship between the molten steel’s impact depth on the mould’s wide face and the immersion depth of the SEN at casting speeds of 0.47 m/min, 0.57 m/min, and 0.67 m/min. The immersion depth of the SEN had a notable influence on the impact depth of the molten steel in the mould. At each casting speed, an immersion depth of 80 mm resulted in the smallest impact depth, while a depth of 140 mm produced the largest impact. This trend indicates that an increase in the SEN immersion depth leads to a deeper impact on the molten steel, which in turn causes the high-temperature reflux zone to shift downward and raises the temperature at the lower part of the mould. These findings align with previous studies [12]. Such conditions hinder the solidification of the shell and the upward movement of impurities. On the other hand, an excessively shallow SEN immersion depth may cause substantial fluctuations in the molten steel’s liquid level, potentially affecting the uniformity of the mould slag layer. Therefore, selecting an optimal immersion depth is crucial for maintaining the quality and stability of the continuous casting process.

**Figure 6:**
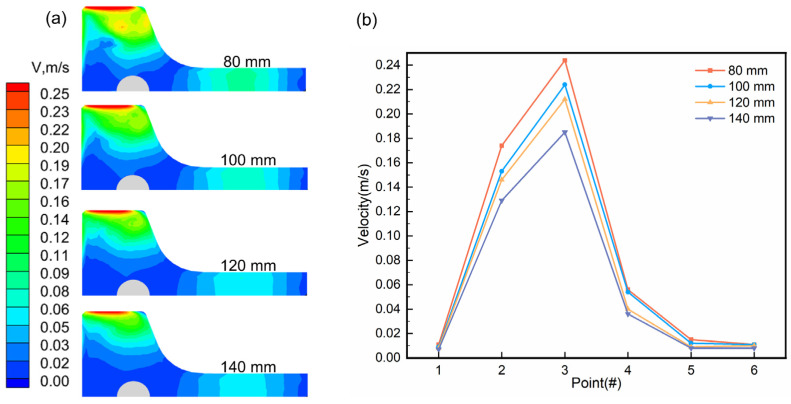
Flow velocity cloud map and velocity distribution at characteristic points on the surface of molten steel at a casting speed of 0.57 m/min and different immersion depths of the SEN. (**a**) Velocity cloud map; (**b**) Surface velocity at characteristic points.

The flow characteristics of molten steel at the meniscus significantly influence the activity and uniformity of slag distribution, which are critical to the production of high-quality beam blanks. A low surface flow rate results in a calm molten steel surface within the mould, hindering the melting of protective slag. Conversely, a high surface flow rate causes excessive agitation of the molten steel surface, expanding the high-flow region and compromising the quality of beam blank casting, slag roll formation, and other related issues [1]. Figure 6 presents the velocity field of molten steel at the mould surface (measured 20 mm below the meniscus) at different SEN immersion depths, as well as the flow velocity distribution at characteristic points for a casting speed of 0.57 m/min. These data were used to assess both the distribution of surface flow velocity and the maximum flow velocity of the molten steel. The highest surface flow velocity was observed near the flange tip (2–3#), attributed to the upward reflux generated by the molten steel impacting the wall and the two ports sprayed onto the flange. In contrast, the flow velocity was lower at the web centre (6#), where the molten steel impacts itself, creating a “self-braking” effect [28]. As the SEN immersion depth increased, the molten steel’s impact depth deepened, reducing the fluctuations at the liquid surface and diminishing its effect on the flow rate. Figure 6b shows that at a casting speed of 0.57 m/min and an SEN immersion depth of 80 mm, the maximum surface velocity reached 0.24 m/s, raising the risk of steel/slag interface damage and slag entrainment [29]. However, at an immersion depth of 120 mm, the maximum surface velocity decreased to approximately 0.2 m/s, which was more favourable for reducing these risks, while maintaining adequate fluidity to support uniform solidification and high-quality beam blanks.

**Figure 7:**
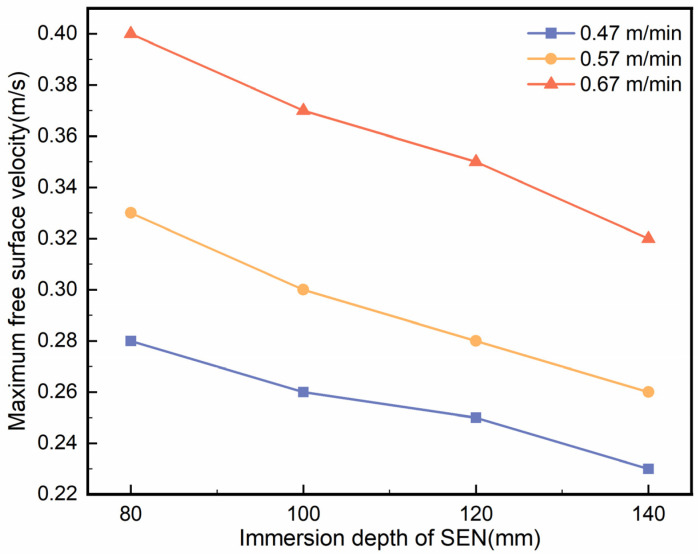
Maximum molten steel flow rate on the mould surface at varying SEN immersion depths.

Figure 7 shows the relationship between the maximum surface flow velocity of the mould and the immersion depth of the SEN at casting speeds of 0.47 m/min, 0.57 m/min, and 0.67 m/min. For each casting speed, the maximum surface flow velocity ranged from 0.2 to 0.4 m/s across different immersion depths. As the SEN immersion depth increased, the maximum surface flow velocity decreased. This trend can be attributed to the deeper immersion of the SEN, which results in a greater impact depth. This, in turn, reduces the backflow of molten steel and mitigates surface fluctuations. For instance, at an immersion depth of 80 mm and a casting speed of 0.67 m/min, the maximum surface flow velocity reached 0.4 m/s, which increased the likelihood of slag entrapment and exposed the beam blank to oxidation. However, when the immersion depth was increased to 140 mm, the maximum surface flow velocity decreased to 0.32 m/s. Thus, increasing the immersion depth reduced the surface flow velocity, mitigating risks such as slag entrapment or steel exposure, which aligns with findings reported in the literature [30].

In the production process, the optimal immersion depth of the SEN at each casting speed is typically not constant but varies within a specific range. Therefore, through a comprehensive analysis of the effects of different immersion depths on the flow field within the mould and considering the casting speed range of 0.47–0.67 m/min, it is important to reduce liquid level fluctuations and ensure a smooth and uniform distribution of surface velocity and velocity gradient [28]. Based on these factors, the recommended immersion depth range for the SEN is 100–120 mm.

### 3.2. Influence of Casting Speed on Flow Behaviour

**Figure 8:**
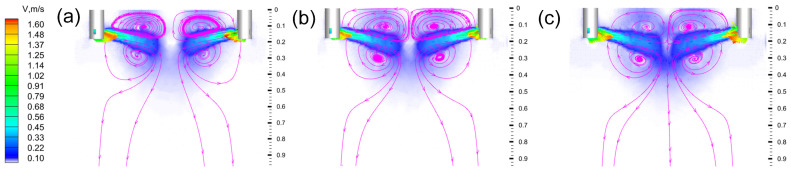
Velocity field and streamline diagram of molten steel on the wide face of mould at different casting speeds: (**a**) 0.47 m/min; (**b**) 0.57 m/min; (**c**) 0.67 m/min.

Building upon the optimal SEN immersion depth identified for this mould, the effect of casting speed on the molten steel flow behaviour within the mould was further investigated. Figure 8 shows the flow field trajectories on the wide face at varying casting speeds, with the SEN immersion depth fixed at 120 mm. The molten steel from the two SENs was directed towards the centre of the web, where it collided, causing part of the molten steel to flow towards the surface, while the remainder moved downward, thereby forming a classic double-roll flow (DRF) pattern within the mould [31]. As the casting speed increased, the flow rate of the molten steel within the mould also increased, resulting in higher jet velocities at the SEN outlets and deeper penetration of the molten steel. Specifically, at casting speeds of 0.47 m/min, 0.57 m/min, and 0.67 m/min, the impact depths on the wide surface of the mould were 269 mm, 305 mm, and 337 mm, respectively.

**Figure 9:**
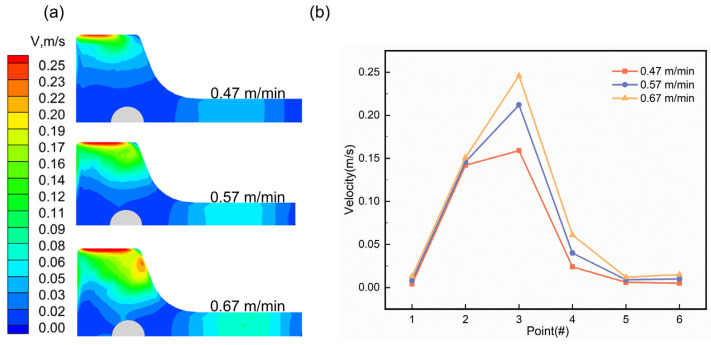
Distribution of free liquid surface velocity of molten steel in the mould at different casting speeds at an immersion depth of 120 mm. (**a**) Velocity cloud map; (**b**) Surface velocity at characteristic points.

Figure 9 shows a cloud diagram of the velocity distribution of the free surface of the molten steel in the mould at different casting speeds and the distribution of the flow velocity at the characteristic points at an immersion depth of 120 mm. Figure 9a shows that due to the direct impact of the molten steel ejected from the side port, the surface flow velocity of the molten steel in the outer corner of the flange (2#) and the centre of the tip of the flange (3#) was large. Upon increasing the casting speed, the flow rate of molten steel in the upper reflux area increased, and the surface flow rate also increased, which increased the surface activity of the mould. During continuous casting, if the molten steel surface flow rate is too large, the probability of the formation of protective slag may also increase. If the surface flow rate is too low, the corresponding slag surface temperature will also be low, which prevents the protective slag from melting, resulting in the uneven solidification of the continuous-casting billet shell. As shown in Figure 9b, when the immersion depth was 120 mm, the flow velocity distribution at the characteristic points also increased as the casting speed increased from 0.47 m/min to 0.57 m/min to 0.67 m/min. In the narrow-face centre (1#), R corner (5#), and web centre (6#) the molten steel either did not directly impact the characteristic points, or the “self-braking” effect made the surface flow velocity in these areas smaller.

The actual production casting speed affects the continuous casting production efficiency. Comprehensive analysis showed that the optimal casting speed range was 0.57–0.67 m/min for an SEN immersion depth of 120 mm.

### 3.3. Verification of Numerical Simulation Results

**Figure 10:**
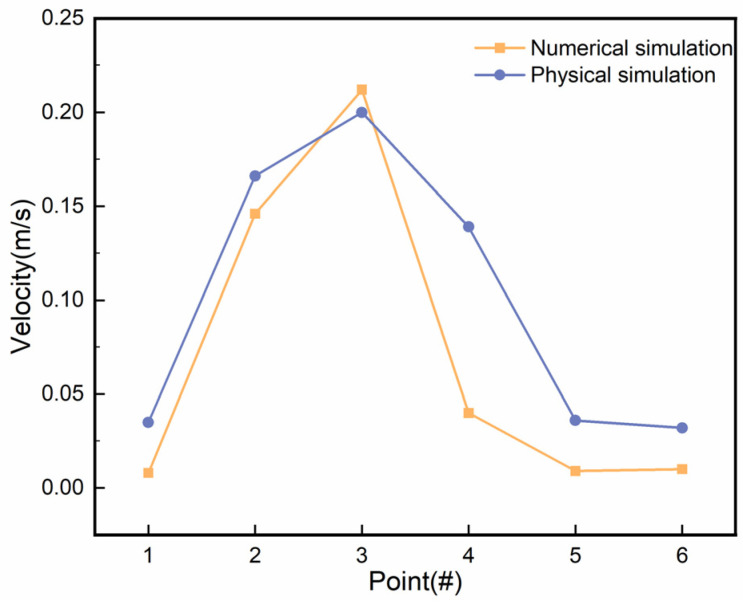
Comparison of the velocity distribution of characteristic points between numerical simulation and physical simulation.

To characterise the flow state of molten steel within the mould and the fluctuations on its surface, as well as to validate the accuracy of the numerical simulation, a physical simulation was conducted. An immersion depth of 120 mm was chosen to study the flow of the molten steel at a casting speed of 0.57 m/min. A comparison of the numerical simulation and physical tests in the mould flow field is shown in Figure 10. Based on the process parameters, the characteristic points at the surface flow velocity distribution were selected. The numerical simulation and physical simulation at the characteristic points of the surface flow velocity distribution trend were consistent, but the limitations of the numerical simulation method required assumptions to simplify the solution process. This also caused the numerical simulation results to deviate from actual results. Due to the limitations of experimental device regulation and measurement instrument precision, the data measured by physical simulation also showed a slight error.

**Figure 11:**
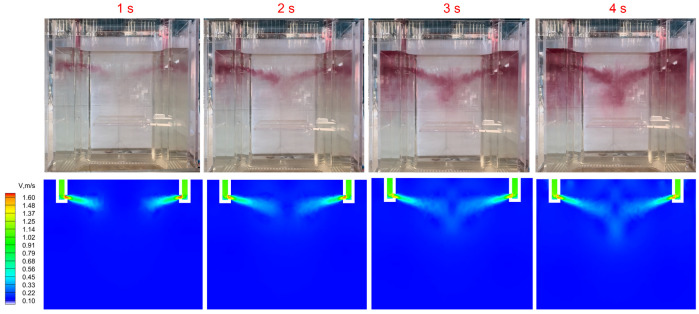
Comparison of flow forms between numerical simulations and physical simulations.

Figure 11 points out the comparison between the physical simulation and numerical simulation with the addition of potassium permanganate tracer dye at an immersion depth of 120 mm and a casting speed of 0.57 m/min. The flow field patterns obtained from both numerical and physical simulations were consistent, with similar impact ranges observed in both cases. The flow form of the numerical simulation was consistent with the flow form and distribution of potassium permanganate tracer dye. Therefore, the flow behaviour of molten steel in the mould under different working conditions studied by numerical simulation was determined to be reasonable.

## 4. Conclusions

In this paper, numerical and physical simulations were used to investigate the effects of the SEN immersion depth and casting speed on the molten steel flow behaviour of a three-port SEN mould specially designed for the continuous casting of beams of ultra-large-section blanks. The key findings were as follows:

(1) The mould flow behaviour was significantly influenced by the immersion depth of the SEN. The impact depth of molten steel in a three-port SEN structure mould increased with the SEN immersion depth, but the overall impact depth was small. Within the SEN immersion depth of 80–140 mm, the impact depth was 230–350 mm. At deeper SEN immersion depths, the upward reflux affected surface fluctuations of the surface, which lowered the surface flow rate.
(2) Upon increasing the casting speed, the impact depth of the molten steel deepened, and the free surface flow velocity and high-velocity zone range also increased. Within the casting speed range of 0.47–0.67 m/min, the maximum surface flow velocity of molten steel was 0.2–0.4 m/s. By selecting an appropriate immersion depth of the SEN, the surface flow rate distribution was uniform, and the activity was better, which promoted the uniform distribution of the protective slag layer and melting.
(3) The flow field distribution patterns in the mould, as observed from both physical and numerical simulations, were found to exhibit consistency, with comparable impact zones and flow characteristics of the molten steel. Numerical simulations provide an accurate, economical, and convenient method to study the molten steel flow behaviour in moulds under different process parameters. Optimising the internal flow characteristics of the mould by selecting optimal process parameters can enhance pouring temperature and solidification uniformity, thereby improving the quality of beam blanks. This ultimately improves the production efficiency of continuous casting.

## Figures and Tables

**Table 1:** Physical performance parameters of steel.

Parameter	Value
Density/$(kg\cdot m^{-3}$)	7200
Viscosity/$({kg\cdot m}^{-1}s^{-1}$)	0.0062
Specific heat/$(J\cdot {kg}^{-1}K^{-1}$)	760
Thermal conductivity/$({W\cdot m}^{-1}K^{-1}$)	32
$\mathrm{Latent}\;\mathrm{heat}/({J\cdot kg}^{-1}$)	274,500
Solidus temperature/(K)	1723
Liquidus temperature/(K)	1790
Molten steel temperature/(K)	1815

**Table 2:** Water model experimental flux values.

$\mathrm{Casting}\;\mathrm{Speed}/(m\cdot {min}^{-1})$	$\mathrm{Flux}/(m^{3}\cdot h^{-1})$
0.47	9.77
0.57	11.85
0.67	13.93

## Data Availability

The original contributions presented in this study are included in the article. Further inquiries can be directed to the corresponding author.
